# What Does a Single-Item Measure of Job Stressfulness Assess?

**DOI:** 10.3390/ijerph16091480

**Published:** 2019-04-26

**Authors:** Jonathan Houdmont, Liza Jachens, Raymond Randall, Sadie Hopson, Sean Nuttall, Stamatia Pamia

**Affiliations:** 1 Centre for Organizational Health and Development, Division of Psychiatry and Applied Psychology, School of Medicine, University of Nottingham, B Floor, Yang Fujia Building, Jubilee Campus, Wollaton Road, Nottingham NG8 1BB, UK; msxsjh@nottingham.ac.uk (S.H.); msxscn@nottingham.ac.uk (S.N.); msxsp10@nottingham.ac.uk (S.P.); 2 Psychology, Sociology and Professional Counselling, Webster University, 1293 Geneva, Switzerland; jachens@webster.ch; 3 Management School, University of Sheffield, Sheffield S10 1FL, UK; r.randall@sheffield.ac.uk

**Keywords:** cognitive interview, psychosocial working conditions, psychological wellbeing, single-item measure, construct validity

## Abstract

Single-item measures of global job stressfulness are increasingly used in occupational health research, yet their construct validity remains unexplored. This study used a qualitative approach to identify frames of reference that underlie self-ratings on such a measure. Data were collected from a convenience sample of 55 adults in full-time employment who completed a single-item measure inviting a rating of the extent to which their job is generally stressful. A cognitive interview schedule was used to explore the factors taken into account when providing a global rating, with thematic analysis applied to identify themes in the interview transcripts. The most common frames of reference were the presence of problematic psychosocial working conditions, particularly job demands. Health characteristics, predominantly poor psychological wellbeing, emerged as a further less dominant secondary theme. Almost half the sample cited four or more referents. In terms of the timeframe under consideration, most participants referred to a long timeframe such as their work in general, with some specifying their current job and, a few, recent weeks. These findings shed light on the frames of reference used to inform judgements on global job stressfulness elicited by a single-item measure and in doing so contribute to the evidence base to support the application of such measures in occupational health research and organisational psychosocial risk management activities.

## 1. Introduction

It has become commonplace in occupational health research and practice for workers to be asked to rate their global job stressfulness on a Likert-type scale that typically ranges from *not at all stressful* to *extremely stressful*. Such measures draw on respondents’ personal understanding of the term ‘stress’ and the degree to which that is experienced in their job. These “crude single-item surrogate indicator(s) of job stressfulness” ([1], p. 14) are useful when the objective is to produce an overall indication of job stressfulness based on respondents’ perceptions of personally salient factors rather than a pre-determined set of characteristics. They have become popular with researchers and practitioners keen to minimise assessment burden and maximise survey response rates. However, the construct validity of such measures remains unexplored. This study explores through interviews the frames of reference that underlie self-ratings of global job stressfulness elicited by a single-item measure.

### 1.1. Associations with Health and Wellbeing

There are two bodies of research that provide some insights into the construct validity of single-item global assessments of job stressfulness. The first of these concerns studies on associations between responses elicited by such a measure and responses to established measures of health and wellbeing. The second concerns studies examining associations between self-reported global job stressfulness on a single-item measure and psychosocial working conditions. Ratings from single-item measures of global job stressfulness have been linked to various measures of health and wellbeing. In relation to common mental disorders, cross-sectional research has shown significantly higher levels of depression, anxiety, and emotional distress among those reporting their job as *very stressful* or *extremely stressful* relative to those reporting their job as *not at all stressful*, *mildly stressful*, or *moderately stressful* [2,3,4]. Longitudinal relationships with depressive symptoms have also been demonstrated [5]. Associations with physical health have also been identified. These include: cardiovascular health [6]; asthma [7]; haemoglobin and globulin [4]; and minor physical symptoms including digestive problems, headache, upper respiratory tract illness, and backache [3]. Longitudinal bidirectional relations with sleep quality have been observed among Dutch employees [8]. In relation to occupational safety outcomes, cross-sectional linkages have been found with minor injuries at work and work-related road traffic accidents [9]. Taken together, these findings suggest that among working populations self-reports of global job stressfulness on a single-item measure provide a useful indicator of the likelihood of experiencing various forms of health impairment.

### 1.2. Associations with Psychosocial Working Conditions

Single-item measures of global job stressfulness are linked to exposure to psychosocial working conditions that can lead to health impairment. For instance, large-scale cross-sectional studies involving civil servants [10] and police officers [11,12] found moderate to strong correlations between responses to a single-item measure of job stressfulness and psychosocial work environment domains assessed in the UK Health and Safety Executive’s Management Standards Indicator Tool (http://www.hse.gov.uk/stress/standards/pdfs/indicatortool.pdf). In these studies coefficients ranged from *r* = 0.28 for role ambiguity and conflict to *r* = 0.65 for job demands. In a cross-sectional study of school head teachers similar relationships were observed with generic and occupation-specific psychosocial work characteristics [13,14]. Similar results have been obtained from large representative samples drawn from electoral registers, with associations evident between single-item measures and constructs contained in the Demand-Control [15] and Effort-Reward Imbalance [16] work environment models [2,17]. At the macro level, associations have been observed between scores on a single-item measure of job stressfulness and national economic health [10,18]. Global self-ratings have also been found to mediate links between psychosocial working conditions and both mental health [19] and intention to leave the organization [11]. These findings suggest that evaluations made when responding to single-item measures of global job stressfulness are particularly useful in capturing data on the stressors that impact on individual- and organizational-level wellbeing.

### 1.3. Single-item Measures and Psychosocial Risk Management

The literature review above suggests that responses to a single-item measure of global job stressfulness may provide a useful shorthand indication of likely exposure to problematic psychosocial working conditions and impaired health. That being the case such a measure has important potential application in organisational psychosocial risk management activities. In order to support the application of a single-item measure of global job stressfulness in such activities it is important to first establish its construct validity. A key strength of the single-item approach is that it permits respondents to consider personally salient factors in their job in order to arrive at an overall rating, whereas multi-item measures examine factors pre-determined by the researcher. However, this strength is also problematic as the data generated provide little insight into the private psychological processes that take place to arrive at a publicly disclosed rating. This means that construct validity remains uncertain, casting doubt on whether data from a single-item measure indicates the need for workplace stressor reduction and health promotion intervention.

Clarity on the construct validity of a single-item measure of global job stressfulness would benefit organisational psychosocial risk management activities in two specific ways. First, it could help inform the focus of in-depth psychosocial risk assessment activity undertaken in response to reports of high job stressfulness on a single-item measure. For instance, if it were the case that a restricted range of psychosocial work characteristics acted as the primary referents when self-assessing global job stress this would indicate that reports of high job stressfulness on a single-item measure could be used to trigger further in-depth psychosocial risk assessment focused on those specific characteristics. Second, knowledge on the construct validity of such a measure could help the targeting of job stress reduction interventions: Knowledge concerning key job stressor referents would empower researchers and practitioners to address these characteristics through intervention. Moreover, it would also provide an evidence base to support the use of a single-item measure of global job stressfulness as an outcome measure for assessing the effectiveness of psychosocial risk reduction interventions.

### 1.4. Aims of the Study

In response to the imperatives outlined above the aim of this study is to investigate the construct validity of a single-item measure of global job stressfulness. Specifically, using a sample of adult workers we use cognitive interviews to explore the frames of reference that underlie self-ratings of global job stressfulness and in doing so set out to answer the question ‘what does a single-item measure of global job stressfulness measure?’

## 2. Method

### 2.1. Participants

The study protocol was developed by JH with ethical approval granted by the Division of Psychiatry and Applied Psychology Research Ethics Sub-Committee at the University of Nottingham (ref. 1089). Data collection was undertaken by SH, SN, and SP in fulfilment of the research component of a Masters Degree in Workplace Health and Wellbeing. A convenience sample of participants was drawn from these individuals’ professional networks via advertisements posted to Facebook and LinkedIn networking groups. A convenience sample was considered appropriate in view of the exploratory nature of this preliminary study. Eligibility criteria included (a) aged 18 or over, (b) in full time employment, and (c) English as a first language or the working language of the participant’s place of employment. The call for participants invited expressions of interest by email. A detailed participant information sheet and consent form was sent following an expression of interest; upon return of a completed consent form arrangements were made for an interview via telephone or Skype. All interviews were conducted in 2018.

In a homogenous group, thematic saturation (the point where additional data collection yields no new information) may be achieved with qualitative data from as few as 12 participants [20,21]. The heterogeneity of workers in our sample was designed to allow quantification of the prevalence of themes in the data. In order to make meaningful comparisons on the proportion of respondents reporting each theme, each researcher attempted to secure no fewer than 15 interviews. This target was exceeded, resulting in a total sample size of 55 participants.

### 2.2. Procedure and Measures

Quantitative studies of construct validity are limited because “the scales and items used as predictor variables are based on assumptions about the dimensions or factors that are important in shaping people’s evaluations … Consequently, with this type of approach, dimensions or factors not captured by the measure are not included in the analysis” ([22], p. 147). The absence of evidence on factors that workers take into account when rating their global job stressfulness rendered such an approach impractical. In this preliminary study we used a qualitative interview methodology to capture the potentially wide array of factors that were salient to a heterogeneous sample. 

The interviews with participants were guided by a schedule of questions. Participants were first asked to provide a global rating of their job stressfulness on a single-item measure developed for the Bristol Stress and Health at Work (SHAW) study [2,3,4] and subsequently used by the UK government in its annual Psychosocial Working Conditions Survey [1]. The measure has been widely used in job stress research [7,9,10,11,12,13,14,17,23] and involves the stem question, “In general, how do you find your job?” with five response options: 1 = *not at all stressful*, 2 = *mildly stressful*, 3 = *moderately stressful*, 4 = *very stressful*, 5 *extremely stressful*.

The single-item measure made explicit reference to job stress. In this way it was distinct from single-item measures that require respondents to consider their global stress, with reports assumed to encompass stress experienced in life domains including but not limited to work. For instance, a rating on a 5-point Likert-type scale may be provided in response to the question: ‘*Stress means a situation in which a person feels tension, uneasiness, nervousness or anxiety, or is unable to sleep at nights due to disturbing thoughts all the time. Do you currently experience this type of stress?*’ [24]. Other single-item global stress measures exist that ask respondents to consider stress ‘*at home and at work*’ [25]. It has been argued that such measures are appropriate for the assessment of job stress on the basis that (a) global stress will affect work outcomes, (b) if applied in workplace setting the measure will be viewed by respondents as relevant to work, and (c) single-item global stress scores tend to correlate with psychosocial work environment perceptions [26]. However, these are likely to activate multiple salient frames of reference (e.g., work, home, family, friends, relationships etc.).

Administration of the single-item measure of global job stressfulness was immediately followed by two questions to explore the frames of reference underlying participants’ responses. These questions were informed by qualitative studies exploring the frames of reference used when providing a rating of self-rated global health [27,28,29,30,31] and oral health [32]. The first question was “What were you thinking about when you answered [insert response] to this question?” This question relates to the first cognitive task involved in providing a response to a question, namely comprehension [33]. The second question was designed to explore the referent timeframe: “When answering the question, did you think about a specific timeframe or period?” Non-directive open-ended verbal probing [33] was then used to obtain further detail. We also recorded participants’ age, gender, and nationality. The mean interview length was 18 min (range = 13–25 min) and interviews were audio recorded using a digital voice recorder. In order to facilitate coding and analysis recordings were transcribed verbatim and entered into NVivo for analysis.

### 2.3. Data Analysis

To identify the frames of reference transcripts were analysed using Braun and Clarke’s [34] six-stage method of thematic analysis: data familiarisation, initial coding generation, search for themes based on initial coding, review of themes, theme definition and labelling, and report writing. Data coding and analysis was limited to participants’ spontaneous responses to the two open-ended questions plus their responses to non-directive probes. Initial coding of the full set of transcripts was undertaken by LJ. JH and RR each independently coded a sub-set of transcripts and identified largely identical codes; in the small number of cases where there was inconsistency, discussion between LJ, JH, and RR helped to clarify themes and reach consensus on codes and their definition and labelling. The focus of analysis was on recurrent themes and sub-themes. In the development of themes we used an inductive process; themes and descriptors were iteratively generated to accommodate the data if existing themes were inadequate or better grouped within a higher theme. Subsequently, themes were collapsed into a manageable number based on commonality of content. Referents mentioned by five or more participants (≥9% of the sample) were considered themes.

Locker et al. [32] noted that qualitative studies investigating frames of reference typically report respondents’ first statement as their frame of reference, pointing out that this ‘first mention’ approach “leads to a substantial loss of information and masks the complexity and multilayered character of health ratings” (p. 79). We therefore included all mentioned frames of reference in our analyses. In order to identify the relative importance of each we performed content analysis by counting the frequency of mentions of each frame of reference.

Following Smith et al. [4] global job stress scores were dichotomized into a low job stress group (responses of *not at all stressful, mildly stressful, moderately stressful*) and a high stress group (responses of *very stressful, extremely stressful*). Smith and colleagues justified the placement of the cut-off point here on two grounds. “First, we consider that no organisation would want their employees to be very stressed. Second, it is common practice to define your ‘high’ group as the upper quartile, and our estimate of the prevalence of perceived occupational stress falls close to this figure” (p. 212).

## 3. Results

### 3.1. Response and Participant Characteristics 

A total of 55 individuals participated in the study. Females comprised 56% of the sample (*n* = 31) and males 44% (*n* = 24). The age distribution was 18–29, *n* = 16 (29%); 30–39, *n* = 22 (40%); 40–49, *n* = 15 (27%); 50–59, *n* = 2 (4%).

### 3.2. Self-rated Job Stressfulness

The distribution of self-ratings of global job stressfulness was *not at all stressful*, *n* = 5 (9%); *mildly stressful*, *n* = 13 (24%); *moderately stressful*, *n* = 25 (45%); *very stressful*, *n* = 12 (22%); *extremely stressful*, *n* = 0 (0%). High job stress was reported by 29% of females and 13% of males, with an overall rate of 22%. These rates are typical of those obtained in studies using the same measure. For instance, among a large sample of 9913 civil servants in Northern Ireland, Houdmont et al. [10] found an overall high job stress rate of 26%, with most of these individuals reporting that their job was *very stressful* (19%) and a small proportion (7%) reporting *extremely stressful*.

### 3.3. Frames of Reference for Global Ratings of Job Stressfulness

When asked to explain what they had thought about when rating their global job stressfulness participants were able to give a clear account of their frames of reference. Table 1 illustrates these referents with example quotations from the interview transcripts and details the number and proportion of participants that identified each. Thematic analysis revealed 5 themes: (1) job demands, (2) job resources, (3) health, (4) coping, and (5) cognition. Themes are presented within two overarching categories, ‘psychosocial work characteristics’ and ‘individual characteristics’; within each category themes are presented in terms of how frequently they were identified by participants.

The first overarching category, ‘psychosocial work characteristics’, contained the dominant theme that emerged from the transcripts: negative aspects of job demands were referred to by four-fifths (82%) of participants. Within Job Demands-Resources theory job demands are defined as “those physical, psychological, social, or organizational aspects of the job that require sustained physical and/or psychological effort and are therefore associated with certain physiological and/or psychological costs” [35] (p. 274). We found that these distinctions, with the exception of physical demands, provided a useful framework for the demand-related sub-themes. We expanded the range of demand sub-themes to encapsulate personal demands centred on “the requirements that individuals set for their own performance and behavior that force them to invest effort in their work and are therefore associated with physical and psychological costs” [35] (p. 279). The second theme within this category concerned job resources, defined as “physical, psychological, social, or organizational aspects of the job that are functional in achieving work goals, reduce job demands and the associated physiological and psychological costs, or stimulate personal growth, learning, and development” [35] (p. 274). Within this theme two sub-themes were identified; these concerned manifestations of support at work and control over how and when work is done. 

The second overarching though less dominant category, ‘individual characteristics’, contained the second most prevalent theme evident in the interview transcripts—health—which constituted a referent for 62% of participants. This category contained two further secondary infrequent themes: coping (reported by 24% of participants) and cognition centred on rumination on work (15%).

### 3.4. Diversity of Referents

Participants’ accounts varied in terms of the number of referents. Respondents typically listed referents that were coded across multiple sub-themes within a single top-level theme: The mean number of codes allocated was 3.22 and 25 (45%) required four codes or more. For instance, many participants listed several aspects of job demand as key referents: *“Amount of hours spent working, decision making and risk to operatives on site because of my decisions. It is my responsibility if anything happens so that is stressful.”* (SH1, M, 35). Similarly, another participant observed, *“Different factors, the amount of samples we receive, the amount of phone calls and emails we receive, what the clients are like and how they speak to me”* (SH2, F, 22). Eight participants (14.5%) referred to only one characteristic and required only one code, 11 (20%) required two codes, and 11 (20%) required three codes. 

### 3.5. Timeframe

In response to the question concerning the timeframe taken into account when providing a response to the single-item global job stress measure the majority of participants (57%) indicated they had considered their work in general without being able to specify a timeframe: *“Just generally, like on average thinking about how stressful my job is. I wasn’t thinking about the last week or anything, just thinking about in general”* (SH1, M, 35). Similarly, *“In terms of how long with work stress it’s been the whole time I have been here so I guess six years. So that’s what I was thinking about, that whole time, as in that is my experience of the job”* (SH3, M, 30). Several participants indicated that they restricted the timeframe to consideration to their current job: *“I have been in the job for three years so I was thinking about that particular period, as that is, I guess, the best way for me to reflect on my job stress or how stressful it is rather than looking at a, like moment in time or one off”* (SH19, F, 38).

A minority of participants (15%) based their rating on a period of time that has been particularly stressful: *“The last year, well January through until about September. I was working a lot of hours during that period and the residents we had at the time were very high maintenance and I think I was quite stressed out of work so when you spend ages at work and for long periods of time”* (SH7, F, 24). A small proportion of participants (9%) restricted their consideration to the last few weeks: *“The last few weeks, I tend to forget about things that have happened before that as there is always so much to think about”* (SH16, F, 35). Two participants (4%) took a critical incident perspective on their job stress in which they reflected on a single particularly stressful day in the recent past: *“I was thinking about a time recently. It was a day a few months ago that was really busy, we had loads of samples in and clients kept calling”* (SH2, F, 22).

## 4. Discussion

### 4.1. Summary of Findings

Our preliminary examination of the construct validity of a single-item self-report measure of global job stressfulness showed that there is some between-participant heterogeneity in terms of the type and number of referents and the timeframe. However, there were many common referents, particularly job demands. Affect and symptoms, predominantly symptoms of poor psychological wellbeing, emerged as a further dominant theme. Accounts varied in terms of complexity, with almost half the sample citing four or more referents. In terms of the timeframe under consideration, most participants referred to an extended period of time rather than just very recent experiences.

### 4.2. Findings in Relation to the Existing Literature 

In our study the emergent themes divided into negative psychosocial work characteristics and health categories, with some participants focusing their responses on just one of these categories, while most referred to both. In this regard our findings are broadly consistent with those of Kinman and Jones [36] who interviewed a sample of working adults to explore what they understood by the term ‘occupational stress’. That study identified a host of distinct and overlapping frames of reference, with 33% of respondents conceptualising the construct as a stimulus specifically related to negative working conditions, 20% as a physical and/or mental response, and 47% as a stimulus-response relationship. Further, in our study there was a high degree of overlap between the negative psychosocial working conditions identified as frames of reference and the list of perceived organizational stressors generated in Kinman and Jones’ study in response to the question *“What are the most stressful aspects of work?”*, with all seven of the frames of reference identified in our study similarly identified as a theme in that of Kinman and Jones. The compatibility of our findings with those of Kinman and Jones indicates that in response to a single-item measure of global job stressfulness workers tap into a common and stable set of referents. The negative psychosocial working conditions identified in our study are also consistent with those of a review of the qualitative literature on job stress that highlighted, among others, interpersonal conflict and work overload among the most important job stressors reported by study participants [37]. It is clear that frames of reference used when considering global job stress are in the most part multifaceted but often influenced by similar stressors. 

Many of the frames of reference identified in our study concerned problematic psychosocial working conditions. Therefore it may be that self-ratings of global job stressfulness provide an efficient proxy measure of threat or harm-loss appraisals [38]. It is reasonable, therefore, to expect that self-ratings of global job stressfulness might demonstrate links with health and provide an indication of the likelihood of poor health in working populations. Support for this prediction can be found in research showing significant differences between those reporting low job stress (reports of *not at all stressful, mildly stressful, moderately stressful*) and high job stress (reports of *very stressful, extremely stressful*) in terms of concurrently self-reported depression and physiological markers [4]. Evidence of the predictive validity of single-item measures of global job stress can also be found in the results of the German Ageing Survey (a nationally representative cross-sectional and longitudinal survey of the German population aged over 40 that takes place every six years): analyses of data collected between 2002 and 2014 showed that an increase in ratings on a single-item measure of global job stress predicted an increase in depressive symptoms [5]. Similar relationships were observed in a Swedish study involving a single-item measure of general stress (non-job-specific) and depression [26]. These findings indicate that single-item measures of global job stressfulness could be used to identify probable cases of common mental disorder in working populations; more research is needed to test the relative sensitivity and specificity of such measures compared to established screening and diagnostic instruments. 

The identification of job demands as the primary referent in our study, allied with findings from previous research showing strong correlations between responses to a single-item measure of global job stressfulness and perceived job demands [10,11,12], might suggest that a single-item measure of job demands could offer an effective alternative to a single-item measure of global job stressfulness in organisational psychosocial risk management activities. Further research could usefully compare the predictive validity of these approaches in order to explore which might best account for workers’ health and wellbeing and, by extension, offer an effective ‘light touch’ psychosocial risk assessment instrument. 

### 4.3. Practical Implications

In our study psychosocial working conditions were the dominant frame of reference for self-reports of global job stressfulness elicited via a single-item measure. These are often modifiable either through action by the employer or self-initiated changes made by the employee [39]. Thus, reports of high job stressfulness on a single-item measure can be viewed as indicative of problematic exposure to psychosocial working conditions that left unchanged might lead to impairment to health. Reports of high job stressfulness on a single-item measure might be used to identify groups of workers that could benefit from stress-reduction interventions and act as a starting point for more detailed analysis of stressors. Reports might also be used to initiate employee-driven job crafting activities to reduce exposure to undesirable working conditions. Although job demands were identified as the dominant referent in this study, symptoms of poor psychological wellbeing were identified as a second dominant theme and not all participants referred to job demands (though most did). This suggests there is a degree of heterogeneity in understanding of the term ‘job stressfulness’ and that it should not be automatically assumed that reports of high job stressfulness indicate exposure to problematic job demands. Nevertheless, the findings of this study allied with those outlined in the literature review suggest that reports of high job stressfulness might usefully trigger further investigation of causes and consequences of stress-related problems as they locally manifest.

Subjective perception of busyness is linked to survey participation rate, with individuals who feel busy being less likely to respond to complete a survey [40], potentially meaning their views are not considered in intervention design. A negative correlation is often observed between survey length and response rate [41,42,43]. These findings highlight the need for short measures of exposure to work stressors in psychosocial risk assessment activities. Shortened measures may be just as valid [44,45]. However, these measures may still contain a relatively large number of items and when applied alongside measures of individual and organizational health should be administered only infrequently if survey fatigue and excessive disruption to work activities is to be avoided. Moreover, application of lengthy surveys to all workers is inefficient when not all worker groups are experiencing stress-related problems. Thus, a single-item measure may offer an efficient first-pass risk assessment to identify groups of workers not exposed to problematic stressors and thus unlikely to benefit from a subsequent more detailed risk assessment. As an efficient first-pass measure of job stressor exposure the single-item measure can be administered at frequent intervals, thereby enabling the early identification of stressor exposures before harm is generated. It may be a way to monitor more frequently the unfolding and long-term effects of stressor-reduction interventions with reduced risk of survey fatigue and high attrition.

### 4.4. Limitations and Further Research

We analysed our data as a whole and did not conduct separate analyses for those reporting high job stressfulness (scores of *very stressful* and *extremely stressful*) and low job stressfulness (scores of *not at all stressful, mildly stressful,* and *moderately stressful*). We made this decision owing to concern that division of the relatively small participant sample might result in failure to identify important themes that were apparent only when analysing the dataset as a whole. This view was reinforced by the uneven distribution of job stressfulness ratings; the high job stressfulness group was small relative to the low job stressfulness group, containing only 9% of participants, none of whom rated their job as *extremely stressful*. Moreover, we took the view that given the exploratory nature of this initial study on the construct validity of a single-item measure of global job stressfulness it was important to avoid the exclusion of referents. However, it may be that different referents apply according to degree of job stressfulness. Studies on self-rated health status have confirmed that those rating their health as unfavourable use different criteria in their self-evaluations to those rating their health in favourable terms. For example, in research concerning self-rating of global health participants who indicated that their health was ‘excellent’ were more likely to draw on positive aspects of their health (such as fitness), whereas those providing alternative responses drew on the presence of ill-health symptoms [31,46]. It is similarly possible, for instance, that workers’ reporting their job as ‘not at all stressful’ might draw on positive aspects of their work rather than focusing on the presence of negative working conditions. Future studies should involve larger samples to permit extensive sub-group analysis according to degree of job stressfulness to identify group-specific referents.

Sub-group analyses might also be useful in respect to socio- and occupational-demographic characteristics such as age and educational level. In our study all but two participants were aged less than 50; it remains unclear whether older workers use different referents. Previous research on self-ratings of global health has suggested that older adults may compare their current health to that at some prior point in time, so that perceived changes may inform current judgements about health [47]. Similarly, age has been identified as a source of variance in frames of reference used by adults in relation to the meaning of oral health [32]. These findings raise the possibility that older workers may differ from younger workers in their approach to assessing job stressfulness, further highlighting the need for research focused on this specific group. Should analyses by socio- and occupational-demographic group identify differences in frames of reference for judgments on job stressfulness this would have important implications for how global ratings should be interpreted and used in occupational health research and practice.

The single-item measure used in our study includes the stem “In general, how do you find your job?” Inclusion of the ‘in general’ prefix might explain why the majority of participants used a frame of reference that was temporally non-specific. It is also notable that some participants reflected on a particularly challenging period in their work, suggesting that for some people the single-item measure of job stressfulness might reflect a maximum from recent experience rather than typical or current levels. Further research is warranted to explore whether the non-specific temporal focus applied by respondents in the current study is an artefact of item wording. Moreover, it would be useful to explore how the framing of a single-item measure of job stressfulness to respondents, particularly when administered in organizational practice, might influence responses. It is possible that the content of any pre-item preamble might have consequences for interpretation of the item. Quantitative research could also establish the degree to which ratings correlate with concurrent health and psychosocial hazard exposures and thereby offer a useful proxy measure of the current status of these characteristics.

Our preliminary findings would also benefit from analyses to explore the relative contribution of identified referents to explaining global ratings of job stressfulness. This approach has proven effective for the development of a comprehensive understanding of frames of reference involved in global self-ratings of other constructs; for instance, oral health [22,32].

This study focused on the first of the four cognitive processes delineated by Tourangeau [48] involved in arriving at a response to a question, namely *comprehension* of the question. Though critical to establishing the construct validity of self-ratings of global job stressfulness, comprehension forms one part of the wider set of cognitive processes that are involved in providing a response to a question. To produce a comprehensive understanding of these cognitive processes further research is required to supplement and integrate research concerning *comprehension* with that addressing additional cognitive processes including *retrieval* of relevant information from memory to answer the question, *manipulation* of retrieved information to make judgments, and *selection* of an answer. These additional processes may become relevant when data are collected by other employees (e.g., line managers or health and safety professionals), especially if workers have concerns about the anonymity of their responses or the way the data might be used. The development of a comprehensive understanding of the cognitive processes involved in providing a rating of global job stressfulness on a self-report single-item scale would go some way to providing an evidence base to supporting the use of such measures in research and practice.

## 5. Conclusions

Recent growth in the use of single-item measures of job stressfulness has outpaced efforts to assess the validity of such measures. We found that the construct validity of a single-item measure of global job stressfulness appears to pivot around the referents of job demands and affect drawn from general experiences. There remains much to be done with many questions unanswered such as whether frames of reference vary by socio- and occupational-demographic characteristics, degree of job stressfulness, and item wording. Further qualitative research examining these questions will provide a valuable foundation for large-scale quantitative research to support the use of single-item self-ratings of job stressfulness as an efficient alternative to multi-item multi-dimensional measures.

## Figures and Tables

**Table 1:** Frames of reference for global ratings of job stressfulness and the number and percentage of participants identifying each referent (*N* = 55).

Referent	Examples	*N* (%)
**Psychosocial work characteristics**		
Demands		45 (82%)
Psychological demands	“I was thinking about the demands of the job and the fact that if I get something wrong then it has big consequences and that’s a lot of pressure.” (SH15, M, 23) “I was thinking about the amount of emotional pressure…due to the nature of the work that I do. It is a 24-hour day constantly thinking about those pressures.” (SH7, F, 24) “If you don’t get things done in a certain timeframe, I think long term you’ll be on the chopping block. Job security is for me probably the biggest stressor.” (SN4, M, 43) “When it comes to events I remember err, periods with very, err close deadlines, with big workload and for feelings I remember frustration, sadness, err, and generally being very intense.” (SP3, F, 27) “There are so many different things you are thinking about at one time. There’s 100 things you have to remember.” (SH4, F, 29)	
Social demands	“I was thinking about customer escalations and new customer appointments…Escalations with a specific customer whereby we had to calm the situation due to them being mis-sold a product.” (SH7, M, 36) “There are so many different nationalities that I work with and they all have their different ways of working and where they’ve come from, and the background they’ve come from, can actually make it quite difficult to understand the way they want to work.” (SN10, M, 48) “We see stressful situations all the time but the feelings I thought about, well that was more about the politics of the job, you know dealing with people, managing every figure and stat more than dealing with the people that we have to treat.” (SH21, M, 35) “Just in general about how the ward can be so busy, about the staff, and about the adult ward and the level of personal care and how mentally unstable they can be” (SH4, F, 29)	
Organizational demands	“I would say the number of hours worked. Because if I was working eight hours a day that would be less stressful. If I would have three hours to relax compared to half an hour or an hour before the kids go to bed that would make a difference.” (SN8, F, 50) “The workload is very high and of course not enough time to, to manage...I have to finish a huge amount of tasks in a very short period of time.” (SP1, F, 31) “I work on my own on a solo response unit so the pressure is on to get there and I am usually the first one to arrive to a patient.” (SH16, F, 35) “I have multiple jobs I have to deal with on a daily basis and each of those have deadlines and different criteria in relation to those jobs and criteria that I have to fulfil...mainly the feeling of trying to manage my time efficiently and thinking about time management in situ against doing the job well.” (SH6, M, 27) “The amount of paperwork I have, the emails I get.” (SH2, F, 22) “The change has been a big deal and we are all flailing in the dark very dismally trying to manage with this new way of working.” (SH5, F, 55)	
Personal demands	“The pressure that you receive from trying to perform and do your job to the best ability I suppose, and if you feel that you’re not making that grade it can be something that would reflect on your stress levels.” (SN10, M, 48) “I want to give out good work and if I can’t do that then it’s stressing me” (SP15, F, 27) “The amount of pressure you place on yourself about your work.” (SH16, F, 35) “The amount of pressure put on me by myself and others to provide the results.” (SH19, F, 38)	
Resources		23 (42%)
Managerial support	“… I have a really supportive team. Even though my workload can be quite heavy and I have a lot of work, because I have a very supportive team behind me and a really supportive manager, it doesn’t feel really draining and stressful so I picked mildly stressful because I have a strong support network behind me.” (SH11, F, 34) “My previous manager… always started by asking how things are going and how I am feeling and showed an interest in what was going on behind the scenes.” (SH9, M, 42) “Maybe listen better, or give opportunities for you to be able to talk about what your concerns are, or where the problem areas are.” (SN3, F, 35) “If I have a good relationship with my senior manager, and I explain the situation and I can see they’re going to take some of the responsibility away from me or they support me or they would be there in the background, this will make a big difference.” (SN12, F, 48) “I have had managers who didn’t have adequate training in how to manage their staff and their definition of being a manager meant shouting at me and wasn’t really about supporting staff to be the best they can be.” (SH11, F, 34) “Because my employer knows about my condition (bipolar) they have adjusted my job accordingly and that has made a massive huge difference, my manager is aware and also I now can manage my work.” (SH20, F, 48)	
Peer support	“My colleagues intervene to relieve some of my responsibility, because we share our decisions. And this is taking some of my stress out, the sharing of these opinions and decisions.” (SN13, F, 45) “Getting support, having support, having a group of people that can support and that you know that you can work together with to get the job done instead of just feeling that I have to do everything on my own.” (SN9, F, 45) “If I’m getting more support from the people I work with, this will also help reduce my stress levels.” (SN12, F, 48) “Literally stress caused by a job but that could be for a variety of reasons, it could be due to not having a supportive team….” (SH11, F, 34)	
Instrumental support	“When people don’t do what they are supposed to be doing and when they are supposed to be doing it.” (SH5, F, 55) “We are always short staffed, trying to get enough staff, trying to get stuff done, then someone is off sick or on leave.” (SH4, F, 29) “Because the timeframe is so short, maybe split the jobs so that there’s more people to help or maybe more resources, maybe more that sort of thing to make it run smoother.” (SN3, F, 35) “I was probably a bit inexperienced to deal with the level of --- scale of the job I was given” (SN6, F, 40) “Well, there aren’t enough staff, simple as.” (SH21, M, 35)	
Job Control	“Not getting a break, I find that stressful.” (SH7, F, 24) “You feel you can’t actually cope with the workload that’s there …because you can’t get everything under control, you can’t see the wood for the trees.” (SH19, F, 39) “So, there’s issues related to third parties that I don’t have any direct control over; those are the occasions when I would say I had felt stressed…I’ve got it down to how in control I feel.” (SN5, M, 41) “I think that the easiness would be my feeling of calm and feeling of being in control and on top of everything, getting everything done in the time frame and feeling prepared for what I need to do, and that keeps me calm. I feel more controlled.” (SN9, F, 45)	
**Individual characteristics**		
Health		34 (62%)
Affect and emotions	“Because it’s easy to identify feelings of helplessness or panic or worry. These are the feelings that are there when you feel stressed.” (SN8, F, 49) “I was thinking about the rollercoaster of emotions my job causes me.” (SH25, F, 28) “When you’re stressful usually you don’t like your job …and feel worried and have some anxiety.” (SP7, M, 33) “The anxiety of turning up for your job.” (SH14, F, 38) “If stress was full on then that could lead to a whole host of stuff like anxiety and feeling down and depressed and having bad relationships with your family and friends.” (SN7, F, 44)	
Physical symptoms	“Normally how tired I am.” (SP2, M, 38) “There are physical things that happen, tight muscles, stiff necks or shoulders. Pain, mentally and physically because I believe stress manifests itself in your body, and usually you become ill in some way.” (SN10, F, 45) “Well anxiety, headaches, not sleeping well, increased appetite or decreased, lack of exercise, being too tired or too stressed that you don’t get out and do stuff.” (SN8, F, 45) “You’re sleep deprived, or you will be feeling so lethargic and burned out because of everything you did that day, that you can barely keep your eyes open to bed time, because you are so drained.” (SN15, F, 37) “When you really feel your heart beats, you really feel that something is going on, that your body is in a completely different state.” (SP6, M, 26)	
Coping		13 (24%)
Coping style	“No particular situation, just my general feelings about my job and how I cope with it.” (SH19, F, 38) “It’s stress related to the job you’re in and it’s also specific to how you deal with the stress and your personality for coping with it.” (SN12, F, 48) “If I feel stressed at my job that I will go back home and binge-eat unhealthy stuff and drink alcohol in order to relax.” (SP11, F, 28) “I was thinking about, well, I get overwhelmed and have to take a deep breath or go outside.” (SH25, F, 28)	
Cognition		8 (15%)
Work rumination	“I am thinking about how easy it is to switch on or off from work.” (SH9, M, 42) “You think about your work all day long and maybe then during the night…it is filling your life. If I worry about things at the weekend.” (SN13, F, 45) “When it starts (stress) building up then you seem to be starting to think about work, you come from work, you’re thinking about it, you’re sleeping, dreaming about it.” (SN7, M, 48) “Thought processes that become difficult to manage I guess, and then kind of spills out when you walk away from working hours and I suppose how stressed you are determines your ability to deal with things when you are out of that door.” (SH8, F, 34) “How uncomfortable you are, worrying a lot, and thinking a lot about your work” (SH4, F, 29)	
